# Topological Properties of Brain Structural Networks Represent Early Predictive Characteristics for the Occurrence of Bipolar Disorder in Patients With Major Depressive Disorder: A 7-Year Prospective Longitudinal Study

**DOI:** 10.3389/fpsyt.2018.00704

**Published:** 2018-12-20

**Authors:** Haiyan Liu, Ke Zhao, Jiabo Shi, Yu Chen, Zhijian Yao, Qing Lu

**Affiliations:** ^1^Department of Psychiatry, The Affiliated Brain Hospital of Nanjing Medical University, Nanjing, China; ^2^School of Mental Health, Wenzhou Medical University, Wenzhou, China; ^3^School of Biological Sciences & Medical Engineering, Southeast University, Nanjing, China

**Keywords:** bipolar disorder, diffusion tensor imaging, graph theory, brain structural network, follow-up study

## Abstract

Bipolar disorder (BD) and major depressive disorder (MDD) are associated with different brain functional and structural abnormalities, but BD is hard to distinguish from MDD until the first manic or hypomanic episode. The aim of this study was to examine whether the topological properties of the brain structural network could be used to differentiate BD from MDD patients before their first manic/hypomanic episode. Diffusion tensor images were collected from 80 MDD patients and 53 healthy controls (HCs); 78 patients completed the follow-up study lasting 7 years. Among them, 12 patients were converted to BD and 64 patients remained MDD. Topological properties of the brain structural networks at baseline were compared among patients who converted to BD, patients who did not develop BD, and HCs. Patients who converted to BD displayed reduced nodal local efficiency in the left inferior frontal gyrus(IFG) compared with HCs and patients who did not convert to BD. There was no significant difference in the nodal global efficiency among the three groups. The findings suggest that the nodal local efficiency in the left IFG could serve as a potential biomarker to predict the conversion of MDD to BD before the occurrence of the first manic or hypomanic episode.

## Introduction

Affective disorders, such as major depressive disorder (MDD) and bipolar disorder (BD), are highly prevalent, recurrent, and disabling worldwide and have unclear pathophysiology. BD has high morbidity and mortality rates, as well as a higher relapse rate and worse prognosis than MDD. However, it's difficult to make accurate diagnosis of BD in clinical practice because approximately half of BD present with a depressive episode as their first episode (1). BD is often misdiagnosed as MDD when patients are undergoing a depressive episode and without a history of any hypomanic or manic episodes (2). Some patients with MDD may be at a pre-onset stage for BD, and manifests on a later stage as BD. Therefore, the problems of the bipolar spectrum and the continuum of affective disorders demand more attention (3). Approximately 40–70% of patients with BD are misdiagnosed as MDD and only 20% of BD patients who were experiencing a depressive episode were diagnosed with the disorder in their first treatment, the mean delay between illness onset and accurate diagnosis was 5–10 years (4, 5), the greatest risk of transition from MDD to BD in the first 5 years (6).

Depressive symptoms and sub-threshold hypomania commonly exist in both BD and MDD, which is an important reason for the difficulty in differentiating BD from MDD. Misdiagnosing BD as MDD may have great adverse impact, including inappropriate treatments, a high risk of conversion to mania, and poor clinical outcome (7, 8), these problems can be reduced by early, correct and timely diagnosis of BD. Many clinical scales have been developed to distinguish BD from MDD patients. These scales involve the clinical characteristics related to BD, such as sub-threshold hypomania, atypical features, frequent episodes, and family history of BD (9, 10). However, the current scales and risk factors can only predict whether patients with MDD may convert to BD, the structured clinical interview and long term prospective follow-up studies are needed to confirm the utility of the assessment (11). Knowledge of the pathophysiology of BD has progressed rapidly over the past years. Using neuroimaging techniques to identify the biomarkers that differentiate BD from MDD is an important pathway to improve the clinical recognition of BD (12). The objective biomarkers vary between BD and MDD which represent the underlying pathophysiologic processes are expected to provide a basis for the identification of BD.

In recent years, a growing number of neuroimaging studies have reported neural abnormalities in affective disorders, in particular, functional, and structural alterations in the prefrontal and limbic regions (13–17). Most evidence indicates abnormal relationships between the prefrontal cortex and limbic structures, including the orbitofrontal cortex, anterior cingulate gyrus, and the hippocampus in patients with BD and MDD (18–23), which contribute to the emotional dysregulation. The identification of these potential biomarkers could improve the understanding of the characteristics of BD patients during depressive episodes.

Abnormalities in white matter tissue have significant implications for the functioning of the brain (24). Increased deep white matter hyperintensity has been observed in bipolar depression compared with MDD patients (25). As an exciting method that will assist with the identification and the localization of specific lesions that may be correlated with neurobehavioral syndromes, the diffusion tensor imaging (DTI) technique has been used to investigate the white matter structural differences between BD and MDD (26–28). However, few studies have been conducted to directly compare patients with BD and MDD. For example, abnormally reduced fractional anisotropy (FA) in the inferior temporal cortex and anterior callosal fibers, as well as reduced volume in the habenula nucleus have been found in BD vs. MDD patients (28–30). One study found that both MDD and BD patients shared similarities in the brain biochemical abnormalities in the deep white matter (31). Another study suggested that regional gray matter volume should be investigated further as a clinical diagnostic tool to predict BD by MRI and a Support Vector Machine (32). Studies comparing unipolar and bipolar individuals have shown reduced habenula volume, decreased FA in the left posterior cingulum in BD but not MDD, and differential patterns of the functional network, especially in emotion regulation, reward, and attentional control-related neural circuitry in BD vs. MDD depression (33–36).

Advanced understanding of the neuroimaging differences between BD and MDD may help to clarify the neurophysiology of BD. The previews structural and functional neuroimaging studies that directly comparing patients between MDD and BD have showed differences in the limbic neural circuits. BD patients may display more prominent abnormalities than MDD patients in the white matter connecting key regions, such as cingulate cortex, prefrontal cortex, and greater volume reduction and emotion dysregulation in the hippocampus, default mode network, medial prefrontal cortex et al. (33, 34, 36–39). However, MDD is the most onset episode of BD, the long-term course of the illness is dominated by depressive episodes rather than manic symptoms, and the symptoms of mania or hypomania often occur after depressive episodes. The major limitation in our knowledge may be directly comparing BD and MDD at the cross-sectional level, which ignores the influence of “false unipolar disorder,” (2, 8), omits the conversion from MDD to BD, resulting in inconsistency and non-objectivity of results (40).

Based on previous research, we conducted an at least 7-year prospective follow-up study to examine the predictive ability of topological properties of the whole brain structural network for the conversion to BD among MDD patients. We supposed that the BD patients may have brain structural abnormalities before the first manic or hypomanic episode and that the topological properties of brain structural networks may be used to distinguish between patients who would convert to BD and those who would not.

## Materials and Methods

### Participants and Assessments

Eighty participants were recruited from inpatient facilities at the Nanjing Brain Hospital between September 2006 and July 2010, aged 18–55 years with a diagnosis of MDD without a prior history of mania or hypomania. All the patients were clinically diagnosed with MDD according to the DSM-IV criteria, assessed by the general clinical screening and the structured Mini International Neuropsychiatric Interview(M.I.N.I.6.0, Chinese version), had a minimum score of 24 rated with the 17 item HDRS in the baseline. All the patients did not take medicine or underwent a washout period of at least 2 weeks to ensure the psychotropic medications were completely cleared from the body. Fifty-three age-, gender-, and handedness-matched healthy controls (HC) were recruited from those living in the same places or nearby to the patients. The inclusion criteria for HC included M.I.N.I. to confirm the absence of a psychosis history, and the Self-rating Anxiety Scale (SAS) and the Self-rating Depression Scale (SDS) to exclude anxiety and depression; the family history of mental illness was negative.

The exclusion criteria for the study were: (1) left-handedness; (2) disease of the nervous system and any another major psychiatric illness; (3) history of head trauma; (4) serious physical illness or alcohol or drug dependence. All participants were native Han and received MRI scanning within 2 days of initial contact at baseline. Healthy subjects with a family history of major psychiatric or neurological illness in their first-degree relatives were excluded.

### Natural Follow-up

Patients were required to have at least 7 years of bi-annual follow-up care, and to have been diagnosed with MDD or BD at the end of the 7 years by senior experienced psychiatrists. All participants underwent the Structured Clinical Interview or DSM-IV, assessments of the HRSD-17 and Young Mania Rating Scale (YMRS) bi-annually. Seventy-eight patients completed the study; among them, one changed into dementia, one was amended to schizophrenia, 12 were identified as BD, and 64 were initially diagnosed with MDD. The conversion rate to BD was 15.4%. Considering the matching of demographics, such as age and education among the three groups, and data quality problems (noise and artifacts), the final sample comprised 12 patients with BD, 44 patients with MDD, and 37 HC subjects. Details as follows, in the process of extracting the network model, the connectivity of the network may be destroyed, that is, isolated nodes may appear. The appearance of the isolated node will make the shortest path of this node and other nodes become infinite. Among the MDD, eight cases were isolated point data, three cases had a low number of education years, and seven cases had data quality problems. As for the HC controls, three cases had isolated points, eight were graduate students, and five cases involved low data quality with noise and artifacts. The final sample comprised 12 patients with BD, 44 patients with MDD, and 37 HC subjects (Table 1).

**Table 1:** Demographic and clinical data of the BD, MDD, and HC.

**Variable**	**BD**	**MDD**	**HC**	***P***
No. subjects	12	44	37	-
First/recurrence	4/8	20/24		
age (years)	36.92 ± 13.72	39.73 ± 8.94	35.35 ± 11.17	0.17^a^
Gender, male/female	8/4	19/25	14/23	0.21^a^
Education (years)	10.58 ± 2.35	10.86 ± 2.09	11.92 ± 3.09	0.12^a^
Age range (years)	18–51	22–50	23–50	-
Duration of illness (years)	6.97 ± 10.13	5.55 ± 6.89		0.57^b^
HAMD17 score	25.75 ± 1.82	27.48 ± 3.56		0.11^b^
Recurrent number	3.58 ± 1.98	2.32 ± 1.41		0.02^b^
Age at onset	27.42 ± 12.37	34.91 ± 8.89		0.02^b^

Data are presented as mean ± deviation. BD, bipolar disorder before experiencing mania or hypomania at baseline, and transformed bipolar depression during follow-up; MDD, major depressive disorder at baseline and during follow-up.

a The P-value was obtained by ANOVA.

b *The P-value was obtained by two-sample two-tailed t-test*.

Of these 12 BD patients, five cases changed into BD within the first year, five cases in the second year, one case in the fourth year, and one case in the fifth year. Considering the matching problems of the general population such as age, education among three groups,

### Image Acquisition and Data Preprocessing

Both structural MRI and DTI were performed with the Sigma system (1.5T GE Medical Systems Sigma, NV/i), Head motions were minimized with restraining foam pads during scanning. Firstly, T1-weighted images were acquired. T1-weighted axial images: repetition time/echo time (TR/TE) = 500/14 ms, thickness/gap = 1.0/0 mm, inversion time = 400 ms, matrix = 256 × 128, field of view (FOV) = 240 × 240 mm^2^, in-plane resolution = 256 × 192. DTI scans: Diffusion was measured along 25 non-collinear directions (b value = 1000 s/mm^2^), and an additional image without diffusion weighting (i.e., b = 0 s/mm^2^), TR/TE = 10,000 ms/81.2 ms, FOV = 240 mm, Matrix = 128 × 128, NEX = 2, slice thickness = 4 mm without gap. The DTI acquisition time was 9 min and included 780 files for each subject.

Data were processed with the Functional Magnetic Resonance Imaging of the Brain software Library-FMRIB's Diffusion Toolbox (FSL, http://www.fmrib.ox.ac.uk/fsl/fdt/index.html). First, DTI data were corrected for eddy currents and motion artifacts by applying a rigid-body transformation of each diffusion-weighted image to the b0 image. The diffusion tensors at each voxel were estimated according to the Stejskal and Tanner equation. Three eigenvalues and eigenvectors were obtained by diagonalization of the tensor matrix. Then, the FA map was calculated. Each b0 image was registered to the T1-weighted image and then registered to the MNI-152 space. The transformation matrices from diffusion space to MNI space were obtained by the aforementioned two registration steps for the subsequent processing.

### Network Construction and Topological Properties

The network was constructed by defining nodes and estimating edges. The method that we used to define the nodes and edges in each network was similar to the previous studies whose node definitions were based on the automated anatomical labeling template (AAL) with 90 cortical and subcortical regions (41, 42). The white matter tractography was defined with a Diffusion toolkit (http://www.trackvis.org). In particular, each streamline was propagated using the Fiber Assignment by Continuous Tracking (FACT) algorithm (41). The propagation was terminated if either a minimum angle threshold = 50° was violated or a voxel was encountered with FA under 0.2. Two regions were considered structurally connected if at least two fibers with a length <3 mm. We chose the FA value as the edge weight of the network.

We used the brain connectivity toolbox (BCT) to calculate the local and global efficiency (E_loc_ and E_glob_) of the brain structural networks of all the subjects (41). These two parameters can be used to quantify the properties of “small world.” The global efficiency was in terms of the information transmission efficiency, reflecting information transmission capacity of the whole brain network; the higher the efficiency, the faster the rate of information transmission between network nodes. The local efficiency was defined as the average efficiency of the local subgraphs, which reveal local communication in the network (43).

$$\begin{array}{lll} {\text{E}}_{loc\left(i\right)} & = & \frac{1}{NGi\left(NGi-1\right)}\underset{j\neq k\notin G}{\sum }{\frac{1}{1}}_{j,k}\\ {\text{E}}_{glob\left(i\right)} & = & \frac{1}{N\left(N-1\right)}\overset{}{\underset{j\neq k\notin G}{\sum }}\frac{1}{1_{ij}} \end{array}$$

### Statistical Analysis

Demographic data were analyzed using the one-way analysis of variance (ANOVA) by SPSS17.0 software, and the chi-square test for gender. Clinical data were analyzed using two-sample *t*-tests between the BD and MDD groups (Table 1). Then the local and global efficiency of the brain network were compared by covariance analysis among BD, MDD, and HC groups; the effect of age, gender, and education were analyzed as covariates. To address the problem of multiple comparisons in the lobe-network metrics, the false discovery rate (FDR) correction was used with the threshold of *p* = 0.05 (44).

## Results

### Global and Local Efficiency Among BD, MDD, and HC

**Figure 1:**
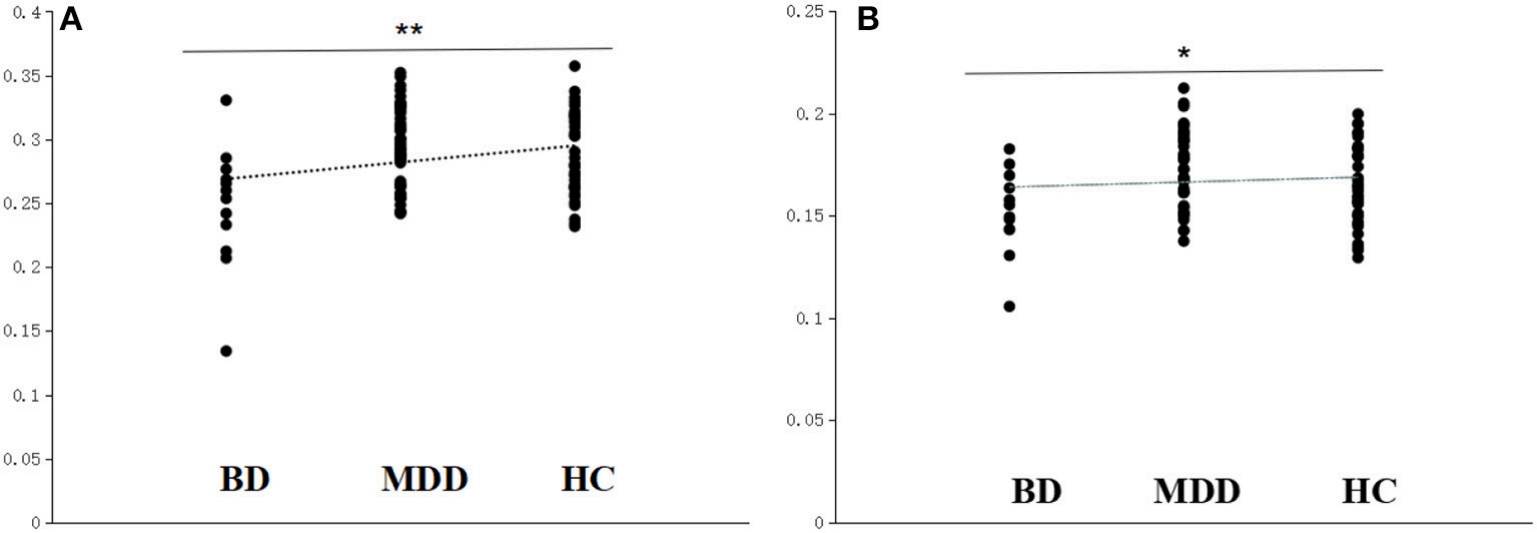
Scatter plots of the local efficiency and global efficiency in the left IFG. **(A)** The local efficiency of the left IFG significantly differed among the BD converters (BD), non-converters (MDD), and healthy controls (HC). **(B)** The global efficiency of the left IFG differed among the BD, MDD, and HC. ^**^Survived critical FDR threshold for multiple comparisons, ^*^P < 0.05, but not surviving FDR correction.

There was no significant difference in the global efficiency of the brain structural network among the three groups although there was a trend to the left IFG (*F* = 6.814 and *P* = 0.003, uncorrected FDR, *P* < 0.05; Figure 1A), and this cluster also extends into adjacent regions, such as the orbitofrontal cortex, triangular part, and precentral gyrus. Significant differences in the local efficiency of the brain structural network were observed among the three groups in the left IFG following the FDR comparison test (*F* = 10.900 and *P* = 0.0004, corrected FDR; Figure 1B). There were also differences in other frontal and insula regions, which were interpreted as trend-level effects (See the Supplementary Materials for detailed results).

### Local Efficiency Between the BD Converters and Non-converters

The local efficiency of the nodes of the networks in BD decreased significantly in the left opercula part of the IFG (*p* = 0.0002, survived critical FDR threshold for multiple comparisons, as described in Table 2).

**Table 2:** Comparison of BD and MDD in terms of local efficiency.

**Brain regions**	**Side**	**BD (*n* = 12)**	**MDD (*n* = 44)**	***p***
		Mean ± SD	Mean ± SD	
Inferior frontal gyrus, opercular part	Left	0.247 ± 0.049	0.031 ± 0.005	0.0002^*^
Putamen	Right	0.279 ± 0.038	0.317 ± 0.029	0.006
Inferior frontal gyrus, opercular part	Right	0.255 ± 0.039	0.289 ± 0.035	0.009
Middle frontal gyrus	Right	0.253 ± 0.061	0.291 ± 0.033	0.009

The p-value was compared using one-way ANOVA and post hoc through LSD and S-N-K and then corrected for multiple testing. Results were FDR-corrected (q = 0.05) over all main analyses.

* *Through FDR*.

Considering the relatively small BD sample, we repeated the comparison many times to validate the result's reliability using a randomized permutation test, and each time we randomly selected 44 MDD patients whose age and gender matched those of the BD patients. Significance was set at a threshold of *P* < 0.05 FDR-corrected. The differences remained significant in the IFG and related areas.

### Local Efficiency Between Patients and HC

Compared with the HC group, there was reduced local efficiency of the left IFG and adjacent regions in the BD group, the similar regions as showing abnormalities in individuals with BD and MDD, but not through FDR correction. No statistically significant differences were found between MDD and HC, and the difference is smaller than that between BD and HC. It is possible that because we applied the whole brain analysis, there was reduced statistical sensitivity due to requiring corrections for the relatively large number of comparisons.

## Discussion

This study was a 7-year longitudinal observation of a cohort of depression patients. Twelve of those patients converted into BD, local efficiency in the left IFG distinguished MDD patients that converted to BD compared to those that did not. Previous studies were mostly cross-sectional studies, and the research focus was on the comparison of patients and healthy individuals or to distinguish between MDD and BD. Long-term longitudinal studies are necessary to test and verify neuroimaging indicators that contribute to the detection of BD. Our findings extend the previous cross-sectional studies that showed the potential impact of IFG in BD, highlighting the deficiencies in the IFG before converting to BD.

Graph theory provides a series of quantitative indicators that can be used to describe the brain structure networks and functional networks. In our previously published article, we found that graph theory methods could help the identification of connectivity without prior model specification (45). Further, graph theory may help to identify biological markers that differ between these disorders, such as the information transfer efficiency of the prefrontal-edge loop being decreased in patients with BD and abnormal hubs of white matter networks in the parieto-frontal cortical circuit contributing to depression discrimination (27, 45, 46). Local and global efficiency are just two of the indicators of graph theory, which is often used to analyze topological changes in the brain structure network of depression.

In our study, we found reduced local efficiency of the left IFG and other frontal and insula regions in BD and MDD compared with HC, which is consistent with previous studies using MRI to explore brain changes in depression. Previous studies concluded that affective disorders were associated with abnormal in the ventral anterior cingulate cortex and prefrontal cortex and their connections with other emotional processing regions including the amygdala, insula, and et al. (47). Some studies in MDD showed white matter structural abnormalities in the dorsolateral prefrontal cortex (DLPFC), such as volume reductions and decreased FA (13, 48–52). The deep white matter hyperintensities (DWMH) indicate a decrease in the density of white matter, such as demyelination atrophy of the neutrophils, and ischemia-associated microangiopathy. MDD had significantly more WMHs than the controls and these were mainly in the frontal and temporal lobes, particularly in individuals with late-onset MDD (26, 53). Specifically, the orbitofrontal cortex and medial prefrontal cortex show *in vivo* and post-mortem tissue loss in both MDD and BD patients, these changes appear to be able to distinguish the BD subjects from the general population (54, 55). It is worth noting that although the trend of reduced local efficiency in patients is consistent with the previous literature, the most significant group differences occurred between the BD and MDD groups, with weaker differences between each patients and the HC group. A similar result was reported in a resting-state fMRI between BD and MDD (56). Our results have not been corrected may be related to the selection of different research indicators, or the uncorrected results reported in earlier research.

Dysfunction of the frontal-subcortical circuits is associated with a variety of neuropsychiatric syndromes (57). As one of the important brain regions, imaging studies have highlighted the importance of the IFG and a meta-analysis demonstrated a spatio-functional diversity of the left IFG for processing of language, working memory, and empathy in healthy humans (58). Decreased functional connectivity from the insular cortex to the DLPFC in depression was found during resting state fMRI, suggests that the weaker causal influence of the insular cortex on the DLPFC might be the pathophysiological mechanism underlying the abnormalities of some cognitive domains in depression (59). BD had extended deficits in a subnetwork containing the left superior and inferior frontal gyrus, postcentral gyrus, and insula (60).

Compared with MDD, we found reduced local efficiency in the left IFG in BD patients before they experienced a manic episode. Similar results were found in global efficiency trend to the left IFG and adjacent regions, but not through FDR correction. These findings hint that patients with BD have brain structural abnormalities before the first manic episode or hypomanic episode and that BD patients would have more abnormalities than MDD patients. Thus, the local efficiency may be a valuable potential identification index measure for distinguishing BD from MDD in the early stages of the disease.

Some neuroimaging studies have directly compared the biological differences between BD and MDD, especially for the white matter and gray matter topological organization, with basically consistent results, suggesting the potential of neuroimaging tools to distinguish between the two disorders. Individuals with MDD and those with BD were differentiated by structural abnormalities in brain regions. Cortical thickness differences were found using MRI between BD depressed patients, MDD patients, and healthy volunteers within the frontal and parietal lobes, and posterior cingulate cortex (23, 61–64). A systematic meta-analysis in pediatric and adolescent depressed subjects found that both MDD and BD presented both shared and distinct impairments in the white matter. More widespread white matter abnormalities were observed in children and adolescents with BD than in those with MDD (65). Structural and functional studies of BD adults supported these results of decreased FA in the left superior and inferior longitudinal fasciculi in BD vs. MDD adults in the inferior temporal cortex (28). The study in adult patients reported that MDD patients had higher DWMH scores than either MDD patients or controls (25, 55). A review also concluded that there were more widespread abnormalities in the white matter connectivity and hyperintensities in BD than MDD, and habenula volume was reduced in BD depression but not MDD (33). Functional connectivity studies in BD patients also showed decreased activity in the insula, temporal, PFC and thalamus compared to MDD during emotional tasks and reward processing (39, 66).

The first graph theory-based brain network analysis study in BD revealed that the bipolar brain networks exhibited abnormal topological properties, such as lower global efficiency relative to healthy controls in 2013 (67). Another DTI study used graph theory to examine the overall topological organization and found that the frontal-thalamo-caudate regions were core neurobiological features associated with MDD (20). At present, there are few studies that report using indicators on global efficiency and the results are not consistent. Complementing our findings, a study on the adaptive brain connectivity features associated with resilience found that the pathophysiology of bipolar disorder is characterized by regional, but not global, abnormalities (68). Another study reported that bipolar I disorder patients exhibit reduced global efficiency (69). In addition, a resting-state fMRI in BD found that the left IFG was functionally impaired (70). A cross-sectional study based on graph theory demonstrated that BDD and MDD differed from each other and HC subjects in density of connections, connectivity path length, and connectivity direction as a function of win or loss anticipation (71). To our knowledge, no long-term follow-up studies using a graph theory approach to compare structural differences between BD and MDD have been reported.

Although differences in the architecture of the brain functional and structural networks between BD and MDD have been reported, whether BD patients already have brain functional or structural changes before their first manic/hypomanic episode is unknown. Our follow-up results suggest a decreased local efficiency in the left IFG, putamen, precentral, and pallidum, and this is mostly prominent in the left IFG. This result supports a previous cross-sectional study and suggests that changes in the topological organization of the cognitive-emotional circuitry and fronto-parietal circuitry may be specific to during the depressive episode. Therefore, depressed individuals with BD may differ from those with MDD in neural mechanisms. On the other hand, recent fMRI studies found that inferior frontal cortical activity decreased in adults with BD across different mood states, especially in the ventrolateral prefrontal cortex (72). Abnormal frontal cortical activation during anticipation of an uncertain reward group may distinguish depressed individuals with BD-I from those with MDD. (35). Our results suggest that there are small-world characteristics of the brain anatomical networks in both patients groups and decreased organization of the brain anatomical network in the BD patients, but the information transmission is impaired in local regions and not in the whole brain, in particular, in the left IFG. Prompt the abnormal transmission ability of structural mainly in the local area, rather than the whole brain. Potential differential diagnostic biomarker could be used to predict BD before they were diagnosed.

Great progress has been made in the past decade through molecular biology, genetics, and neuroimaging. But as a multifaceted disease, modern psychopathology of BD still in an unclear state due in part to the limited methodology of psychopathology. Research use combines clinical assessment scales, simultaneous administered with fMRI data acquisition found activations in the right precentral and postcentral gyrus, left superior frontal gyrus, left occipital pole in MDD, confirmed the possibility of translational cross-validation of a clinical psychological test and fMRI (73). This provide another possibility to elucidating the pathological mechanism of affective disorder.

In conclusion, this 7-year prospective follow-up study aimed to find a predictive biomarker for the occurrence of BD in MDD patients before the first manic or hypomanic episode. The results demonstrate that local efficiency in the left IFG has a high predictive ability for the occurrence of BD in MDD patients. The topological properties of the brain structural networks might be a potential objective marker to distinguish BD from MDD in the early stages.

There are several limitations to this study. First of all, our results showed that the conversion rate of MDD to BD was only 15.4%, which was lower than in previous studies (4, 5, 74). This may be related to the age of patients given that only 10 cases were under the age of 25 at baseline. Epidemiological studies suggest that the age of onset of BD is lower than that of patients with MDD (12). Secondly, we performed a naturalistic observation and the YMRS scores were not assessed when the manic or hypomanic symptoms occurred. Finally, the sample size was relatively small and there was no large independent dataset to make a predictive model to confirm our findings. To identify the reliability of these biomarkers, future study should address those limitations by increasing the sample size and measure from onset of BD during progression and in later stages.

## Ethics Statement

The study was approved by the Research Ethics Review Board of Nanjing Brain Hospital, China. All participants and their supervisors signed informed consent after a full written and verbal explanation of the study. All healthy controls also provided written informed consent.

## Author Contributions

HL performed the data collection and wrote the original draft of the paper. KZ conducted the statistical data analysis and contributed to the editing of the paper. JS and YC contributed to the clinical assessment. QL provided the data analysis technology. ZY contributed to the overall management and supervision of the project, and revised the manuscript.

### Conflict of Interest Statement

The authors declare that the research was conducted in the absence of any commercial or financial relationships that could be construed as a potential conflict of interest.
