# Supplementary material for: Topological Properties of Brain Structural Networks Represent Early Predictive Characteristics for the Occurrence of Bipolar Disorder in Patients With Major Depressive Disorder: A 7-Year Prospective Longitudinal Study
Source: Front Psychiatry. 2018 Dec 20;9:704. doi: 10.3389/fpsyt.2018.00704 (PMC6307456; doi:10.3389/fpsyt.2018.00704)
Supplement: Supplementary file 1 [file Table_1.doc]

**Supplementary material**

Table 1 Global efficiency of the networks among three groups

| Brain regions | Side | BD (n=12) mean ± SD | MDD (n=44) mean ± SD | HC (n=37) mean ± SD | F | *P* |
| --- | --- | --- | --- | --- | --- | --- |
| Inferior frontal gyrus, opercular part | Left | 0.152±0.021 | 0.173±0.018 | 0.165±0.019 | 6.184 | 0.003 |
| Precentral | Right | 0.186±0.023 | 0.209±0.018 | 0.208±0.023 | 5.801 | 0.003 |
| Precentral | Left | 0.178±0.019 | 0.199±0.019 | 0.197±0.022 | 5.136 | 0.008 |
| Postcentral | Right | 0.191±0.024 | 0.208±0.019 | 0.211±0.018 | 4.846 | 0.008 |
| Inferior frontal gyrus, orbital part | Left | 0.140±0.020 | 0.168±0.030 | 0.164±0.031 | 4.600 | 0.009 |

Table 2 Local efficiency of the networks among three groups

| Brain regions | Side | BD(n=12) | MDD(n=44) | HC(n=37) | *F* | *P* |
| --- | --- | --- | --- | --- | --- | --- |
|  |  | Mean ± SD | Mean ± SD | Mean ± SD |  |  |
| Inferior frontal gyrus, opercular part | Left | 0.247±0.049 | 0.294±0.031 | 0.288±0.033 | 10.900 | 0.0004^*^ |
| Putamen | Right | 0.280±0.038 | 0.317±0.030 | 0.310±0.027 | 8.089 | 0.001 |
| Middle frontal gyrus | Right | 0.253±0.061 | 0.291±0.033 | 0.296±0.034 | 6.403 | 0.003 |
| Inferior frontal gyrus, triangular part | Left | 0.249±0.052 | 0.285±0.035 | 0.288±0.033 | 6.072 | 0.003 |
| Middle frontal gyrus, orbital part | Right | 0.234±0.106 | 0.299±0.053 | 0.294±0.057 | 6.094 | 0.003 |
| Postcentral | Right | 0.272±0.035 | 0.299±0.028 | 0.302±0.025 | 6.399 | 0.003 |
| Inferior frontal gyrus, orbital part | Right | 0.255±0.039 | 0.290±0.035 | 0.288±0.033 | 5.314 | 0.007 |
| Precentral | Left | 0.266±0.038 | 0.296±0.028 | 0.295±0.033 | 5253 | 0.007 |
| Middle frontal gyrus | Left | 0.247±0.056 | 0.284±0.036 | 0.282±0.039 | 5.040 | 0.008 |
| Inferior frontal gyrus, orbital part | Right | 0.228±0.054 | 0.250±0.042 | 0.268±0.049 | 4.967 | 0.009 |
| Supplementary motor area | Left | 0.276±0.066 | 0.295±0.042 | 0.314±0.043 | 4.974 | 0.009 |
| Inferior frontal gyrus, triangular part | Right | 0.265±0.056 | 0.293±0.038 | 0.299±0.031 | 4.979 | 0.009 |

The p value was obtained by multivariate analysis of covariance, age, gender, and years of education as covariates

^*^ Survived critical FDR threshold for multiple comparisons
